# “Point by point” source: The Chinese pine plantations in North China by evidence from mtDNA

**DOI:** 10.1002/ece3.11570

**Published:** 2024-06-19

**Authors:** Biao Zhou, Zijie Zhang, Hongjing Zhang, Yupeng Li, Yanguang Ma, Shubin Zhang, Shihui Niu, Yue Li

**Affiliations:** ^1^ National Engineering Research Center of Tree Breeding and Ecological Restoration, Key Laboratory of Genetics and Breeding in Forest Trees and Ornamental Plants, Ministry of Education, The Tree and Ornamental Plant Breeding and Biotechnology Laboratory of National Forestry and Grassland Administration, College of Biological Sciences and Technology Beijing Forestry University Beijing China; ^2^ Hebei Academy of Forestry and Grassland Science Shijiazhuang China; ^3^ Wucheng Seed Orchard, Guandishan State‐Owned Forest Administration of Shanxi Lüliang China; ^4^ The Pinus tabuliformis Seed Orchard Lüliangshan State‐Owned Forest Administration of Shanxi Lüliang China; ^5^ Yixian Forestry Development Service Center Jinzhou China

**Keywords:** Chinese pine (*Pinus tabuliformis* Carr.), domestication, genetic structure, geographic variation, germplasm sources, haplotype, mtDNA

## Abstract

The geographical variation and domestication of tree species are an important part of the theory of forest introduction, and the tracing of the germplasm is the theoretical basis for the establishment of high‐quality plantations. Chinese pine (*Pinus tabuliformis* Carr*.*) is an important native timber tree species widely distributed in northern China, but it is unclear exactly where germplasm of the main Chinese pine plantation populations originated. Here, using two mtDNA markers, we analyzed 796 individuals representing 35 populations (matR marker), and 873 individuals representing 38 populations (nad5‐1 marker) of the major natural and artificial populations in northern China, respectively (Shanxi, Hebei and Liaoning provinces). The results confirmed that the core position of natural SX* populations (“*” means natural population) in the Chinese pine populations of northern China, the genetic diversity of HB and LN plantations was higher than that of natural SX* populations, and there was a large difference in genetic background within the groups of SX* and LN, HB showed the opposite. More importantly, we completed the “point by point” tracing of the HB and LN plantings. The results indicated that almost all HB populations originated from SX* (GDS*, ZTS*, GCS*, and THS*), which resulted in homogeneity of the genetic background of HB populations. Most of germplasm of the LN plantations originated from LN* (ZJS* and WF*), and the other part originated from GDS* (SX*), resulting in the large differences in the genetic background within the LN group. Our results provided a reliable theoretical basis for the scientific allocation, management, and utilization of Chinese pine populations in northern China, and for promoting the high‐quality establishment of Chinese pine plantations.

## Graphical abstract

Based on two mtDNA markers, accurately tracing the germplasm source of Chinese pine plantations in north China. Analyzed the genetic diversity and genetic structure of plantation and natural populations. Confirmed the core position of SX* natural populations.

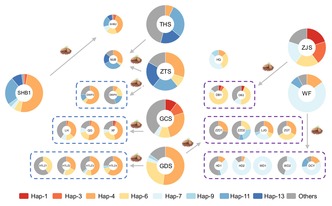

## 1 INTRODUCTION

The domestication of native tree species and the introduction of alien tree species are important contents of modern forestry, which contribute to the development of the modern theory of tree introduction (Wang & Jiang, 1995). Long‐distance migration, geographic variation, and the demonstration mechanism of tree species are important aspects of forest introduction theory and an important basis for biodiversity conservation (Morgenstern, 1996). Understanding the geographical variation and adaptability of tree species, the stability of excellent provenances, and the determination of the suitable growing area of tree species are important scientific issues in the research of genetic breeding of trees (Morgenstern, 1996; Wang & Jiang, 1995; Zenni et al., 2016).

The study of the geographical variation of trees began in the middle of the 18th century, with *Pinus sylvestris* L. serving as the object of research. The results showed that there were general differences in the growth and adaptability of geographic germplasm from different areas of European distribution in French experimental forests, and also identified the geographic germplasm with the highest timber yield (Turnball et al., 1986). The study of geographic variation of forest tree species in China began in the 1980s (Deng et al., 1981; Xu & Fen, 1978), and the seed regions of the major tree species were divided in the 1990s (Chen, 2001). Prior to that, the extent of forest seed allocation in each producing region had not been determined, and there was a generalized cross‐provincial allocation of some tree species for afforestation, making it difficult to achieve high‐quality plantations. More worryingly, seeds from these unidentified plantations and their progeny continue to be used for reforestation purposes, jeopardizing the long‐term survival of tree species (Boshier et al., 2015). The geographical variation and domestication of foreign germplasm at the point of introduction may lead to its gradual domestication into new germplasm adapted to natural growth and cultivation locally. Therefore, it is of great theoretical significance to explore the geographical variation, traceability of germplasm, domestication, and adaptation of plantations, gradually reveal the germplasm source of plantation population, and understand the suitability of foreign germplasm at the introduction site, so as to pursue the correct allocation of plantation germplasm, promote the construction of high‐quality plantations, collect regional germplasm resources, and cultivate plantations (Shang, 2019).

**FIGURE 1:**
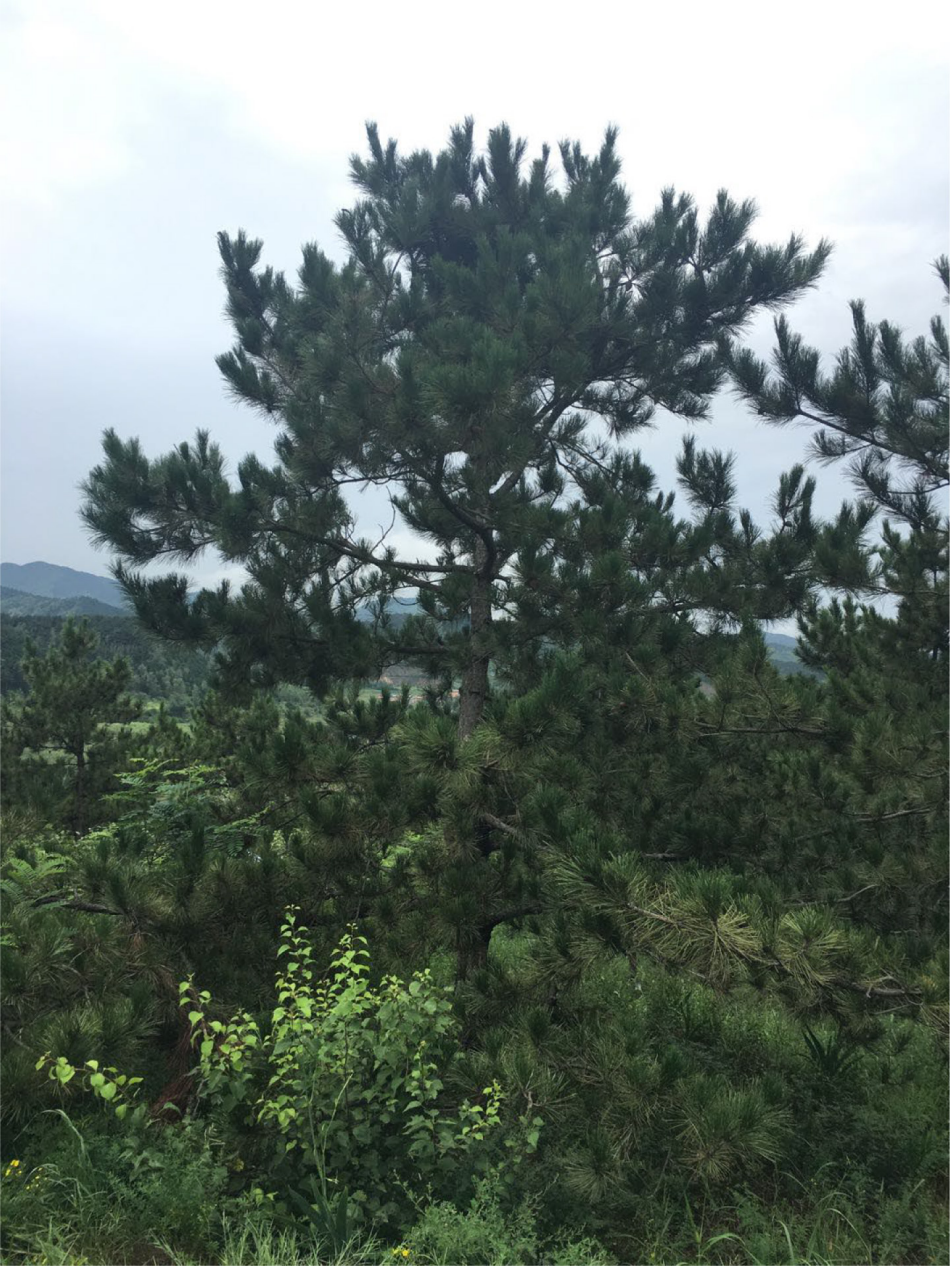
The standard look of Chinese pine.

Chinese pine (*Pinus tabuliformis* Carr.) is an important native timber tree species widely distributed in northern China (Figure 1) (Niu, Li, Bo, et al., 2022; Niu, Li, & Li, 2022). The study on geographical variation (Xu, Guo, et al., 1981; Xu, Sun, et al., 1981; Xu & Tang, 1984) and provenance test (Li, 1983; Wen et al., 1986) of Chinese pine started in 1981, and there were significant differences in adaptability and growth among different provenances, which showed the regional specificity of different provenances (Dong et al., 1991; Liu et al., 2008; Sun et al., 1991; Zhang et al., 2010). Natural and neutral selection were the main reasons for generating the variation in the natural populations of Chinese pine (Meng et al., 2013; Xia et al., 2018). Shanxi Province was the core distribution area of the natural populations of Chinese pine in China, which could be demonstrated by geographical investigation of the natural population distribution in China showing Shanxi Province with the wide distribution of natural population (Fu et al., 1988; Xu, Guo, et al., 1981; Xu, Sun, et al., 1981), and Shanxi Province was the refugees for the Chinese pine population during Last Glacial Maximum (LGM) (Chen et al., 2008; Hao et al., 2018). Due to the prolonged war in modern China and the great demand for wood in the course of the revitalization, the natural forests in northern China, represented by the provinces of Beijing, Hebei, and Liaoning, were excessively deforested. In the 1950s–1960s, large‐scale plantations of Chinese pine trees began in these areas (these stands were mistaken for natural forests in some studies), and the stand archives showed that most of the germplasm came from Shanxi Province, but the exact origin was not clear. Determining the exact germplasm sources of the main Chinese pine plantation populations in northern China could provide a reliable theoretical basis for the scientific allocation, management, and utilization of Chinese pine germplasm, and promote the efficient cultivation of the Chinese pine populations (Boshier et al., 2015; Shang, 2019).

In recent years, we have analyzed the genetic structure and geographical variation of natural populations of Chinese pine in five mountainous areas of Shanxi Province using SSR markers (Wu et al., 2018), and preliminarily understood the genetic relationship between the main plantation populations in Beijing and the natural populations in Shanxi Province (He et al., 2020). Recently, for the first time, we had a more comprehensive understanding of the genetic background of Chinese pine in northern China and found that the natural populations in Shanxi had the status of ancestors in northern China, and “Taiyueshan” was the core distribution area of the natural population of Chinese pine in Shanxi Province, and confirmed that most of the germplasms of Chinese pine plantations in northern China (represented by the provinces of Liaoning and Hebei) could originate from the natural populations of Chinese pine from Shanxi. This indicates that the new adaptive variations and the strong “driving force” of gene flow from the local natural populations have allowed the germplasms from Shanxi to successfully invade Hebei and Liaoning provinces (Zhou et al., 2022). However, due to the limited availability of SSR markers, we could only laboriously determine the exact origin of the germplasm of Chinese pine plantations in northern China, and the “point by point” tracking of the plantation population was still a very difficult task.

Mitochondrial DNA (mtDNA) is characterized by conservative variation (Held & Patel, 2020), which has been widely used to understand the genetic diversity (Uricoechea Patiño et al., 2023), genetic structure, and haplotype characteristics of populations (Liu et al., 2020), determine the evolutionary history (Ye et al., 2019) and geographical origin (Kang et al., 2021) of species, etc. In the genus *Pinus*, mtDNA is maternally inherited and spread via seeds (Neale et al., 1989). It has been widely used as a genetic marker in research to determine the refuge of Chinese pine and *Picea mariana* during the LGM (Chen et al., 2008; Hao et al., 2018; Jaramillo‐Correa et al., 2004), to determine the origin of *Pinus densata* (Wang et al., 2011), the phylogeography of Scots pine in Europe and Asia (Wachowiak et al., 2023), and so on. In this study, 796 individuals representing 35 populations, and 873 individuals representing 38 populations of the major natural and artificial Chinese pine populations in northern China (five groups, including the natural populations of Shanxi Province, SX*, the natural and plantation populations Hebei Province, HB* and HB, the natural and plantation populations of Liaoning Province, LN* and LN) and analyzed by matR and nad5‐1 mtDNA markers, respectively. Our aim was to reveal the genetic diversity, genetic structure, and haplotype traits of five groups and to accurately determine the origin of the germplasm of the HB and LN plantations.

## 2 MATERIALS AND METHODS

### 2.1 Samples and location

**FIGURE 2:**
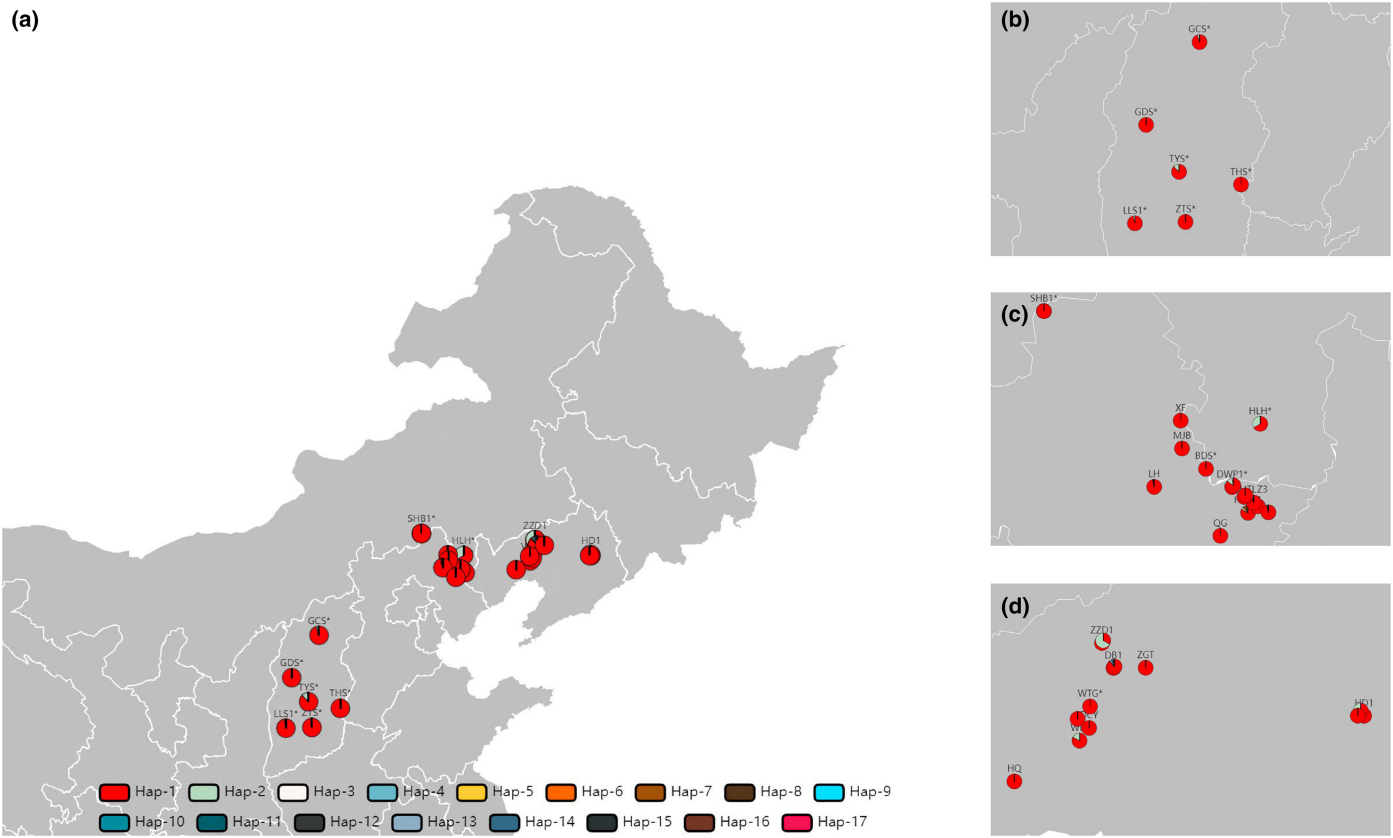
Geographical distribution of haplotypes based on matR marker. (a) All, (b) SX*, (c) HB* and HB, (d) LN* and LN populations.

**FIGURE 3:**
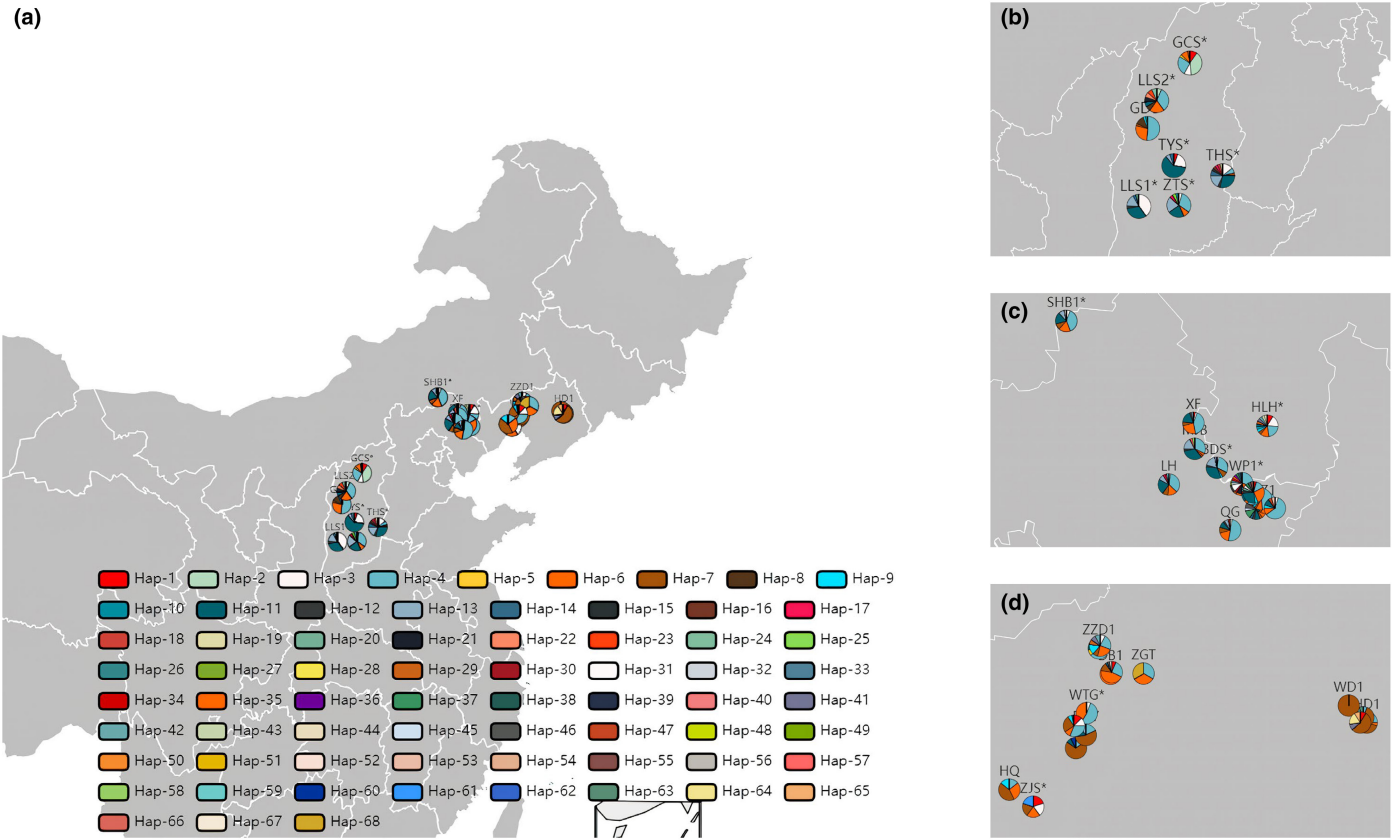
Geographical distribution of haplotypes based on nad5‐1 marker. (a) All, (b) SX*, (c) HB* and HB, (d) LN* and LN populations.

In this study, needles of 796 (873) Chinese pine trees were collected from 35 (38) populations of the major natural and artificial populations in Shanxi, Hebei, and Liaoning provinces in China (Figures 2 and 3, Table S1). We divided all the groups into five groups, including Shanxi Province natural populations (SX*, 7 populations), Hebei Province natural (HB*, 5 populations) and plantation populations (HB, 11 populations), Liaoning Province natural (LN*, 3 populations), and plantation populations (LN, 12 populations), using “*” special representative for the natural population here. More detailed population information is shown in Table S1. About 30–35 individuals (the distance between individuals was greater than 30 m) were randomly selected from each population, and the current‐year Chinese pine needles were collected in 2020 and used for follow‐up experiments.

### 2.2 DNA extraction and concentration identification

Genomic DNA was extracted from the needles using a Plant Genomic DNA Kit (SteadyPure AG21010, Accurate Biotechnology, Changsha, Hunan, China). The quality and quantity of DNA was analyzed by agarose gel electrophoresis. The extracted DNA was quantified spectrophotometrically (Nanodrop 2000c, Thermo Fisher Scientific, Waltham, MA, USA), diluted to 5 ng/μL and cryopreserved at −20°C.

### 2.3 mtDNA markers selection

Based on the selection of mtDNA markers developed by Jaramillo‐Correa (Jaramillo‐Correa et al., 2004; Jaramillo‐Correa & Bousquet, 2003), two mtDNA markers (matR and nad5‐1) were obtained according to the criteria of high polymorphism, strong stability, and good repeatability (Table S2).

### 2.4 PCR amplification and sequencing

The PCR was performed in a reaction volume of 20 μL [1 μL DNA template (5 ng/μL), 1 μL each forward and reverse primer (10 pmol/μL), 7 μL ddH_2_O, and 10 μL 2 × Mei5bio Taq PCR Mix]. The amplification conditions were as follows: 5 min at 95°C, followed by 35 cycles of 30 s at 95°C, 30 s at 58°C (56°C), 30 s at 72°C, with a final extension of 5 min at 72°C, stored at 4°C. The PCR products were tested on a 1.5% agarose gel. Double‐sided sequencing was performed using the ABI 3730 sequencer.

### 2.5 Data analysis

ContigExpress was used for stitching and manual correction of double‐ended sequencing data. BioEdit was used for sequence alignment and clipping (the anterior and posterior 50 bp sequences), and the alignment and clipped sequences were used for subsequent data analysis. The raw sequences of all samples at the matR and nad5‐1 markers were uploaded as the additional information (raw sequencing data).

Genetic diversity parameters, including the number of polymorphisms (*S*), the number of haplotypes (*h*), haplotype diversity (Hd), nucleotide diversity (Pi) and the average number of nucleotide differences (*K*), and indel diversity (*k*(*i*)) were calculated using DnaSP (Librado & Rozas, 2009). Analysis of molecular variance (AMOVA), Tajim's *D*, Fu's *F*s, and the corresponding *p* values were determined using Arlequin software (Excoffier & Lischer, 2010). Statistical analysis using SPSS. MEGA X (Sudhir et al., 2018) was used to construct the neighbor‐joining tree. GenAIEx 6.5 (Peakall & Smouse, 2012) was used to calculate the genetic distance between all populations. STRUCTURE (Falush et al., 2003, 2007; Hubisz et al., 2009; Pritchard et al., 2000) was used to determine the best subpopulations of all populations. A burn‐in period of 100,000 and 100,000 MCMC (Markov Chain Monte Carlo) replicates after burn‐in were determined using an admixture model. The *K* value ranges from 1 to 10, and 10 independent operations were performed for each *K* value. Subsequently, Structure Harvester (Earl & Vonholdt, 2012; Evanno et al., 2005), CLUMPP v1.1 (Rosenberg, 2007), and DISTRUCT (Rosenberg, 2004) were used sequentially to determine the best K, match the clustering results and visualize the results.

The six conserved domain fragments (https://meme‐suite.org/meme/tools/meme) of the two marker sites were extracted and integrated to simplify the number of haplotypes. PopART (Leigh & Bryant, 2015) was used to create the TCS network.

## 3 RESULTS

### 3.1 Genetic diversity

**FIGURE 4:**
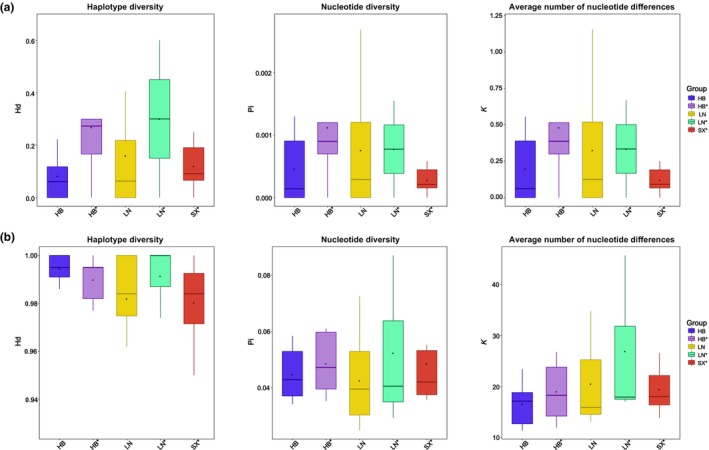
Genetic diversity (haplotype diversity, nucleotide diversity, and average number of nucleotide differences) of sequence based on matR (a) and nad5‐1 (b) markers.

In total, 440 base pair fragments (after sequence alignment and clipping) of the matR marker were generated from 796 sequences originating from 35 populations. Analysis of the sequences resulted in 44 polymorphic sites and 28 (based on full length)/17 (based on six domains) haplotypes. The Hd ranges from (0‐) 0.059 to 0.667, the nucleotide diversity (Pi) from (0‐) 0.00014 to 0.00280, the average number of nucleotide differences (k) from 0.059 to 1.200, and the indel diversity (k(i)) from 0.228 to 1.333 (Figure 4a, Table S3). The comparison of the average Pi between the five groups was HB* (0.00112) > LN > HB > SX* > LN*(0.00023) (Figure 4a, Table S3), indicating that the HB* group had the highest genetic diversity at the matR marker (there was no significant difference between the two pairs). The Tajima's *D* and Fu's Fs values of most populations did not deviate significantly from 0, indicating that the matR marker is a neutral locus and has not been mutated by the environment (Table S3).

In total, 999 base pair fragments (after sequence alignment and clipping) of the nad5‐1 marker were generated by 873 sequences from 38 populations. Analysis of the sequences resulted in 88 polymorphic sites and 225 (based on full length) /68 (based on six domains) haplotypes. The Hd ranges from 0.927 to 1.000, the nucleotide diversity (Pi) from 0.02488 to 0.08705, the average number of nucleotide differences (*k*) from 11.41200 to 45.70000, and the indel diversity (*k*(*i*)) from 52.074 to 135.200 (Figure 4b, Table S4). The comparison of average Pi between the five groups was LN*(0.05233) > HB* > SX* > HB > LN(0.04251) (Figure 4b, Table S4), indicating that the LN* group had the highest genetic diversity at the nad5‐1 marker (there was no significant difference between the two pairs). The Tajima's *D* values of most populations did not deviate significantly from 0, and the Fu's *F*s values showed the opposite path. Under the premise of considering only the Tajima's *D* value, the nad5‐1 marker is also a neutral locus (Table S4).

### 3.2 Genetic structure

**FIGURE 5:**
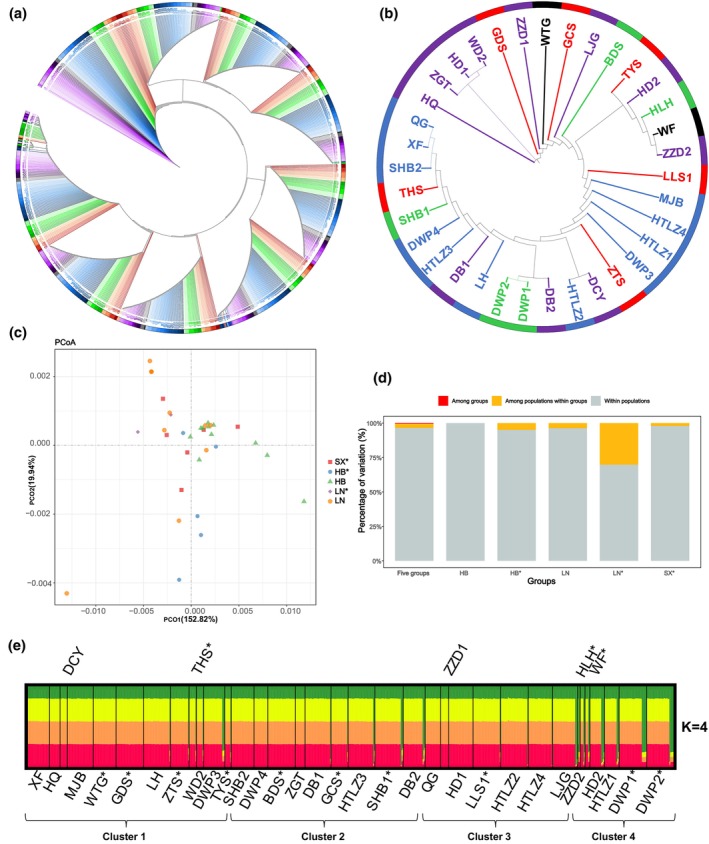
Genetic structure based on matR marker. (a, b) The NJ tree (red, green, blue, black, and purple represent SX*, HB*, HB, LN* and LN group, respectively, different transparencies represent different populations), (c) PCoA, (d) AMOVA, and (e) STRUCTURE results of 796 individuals (35 populations).

**FIGURE 6:**
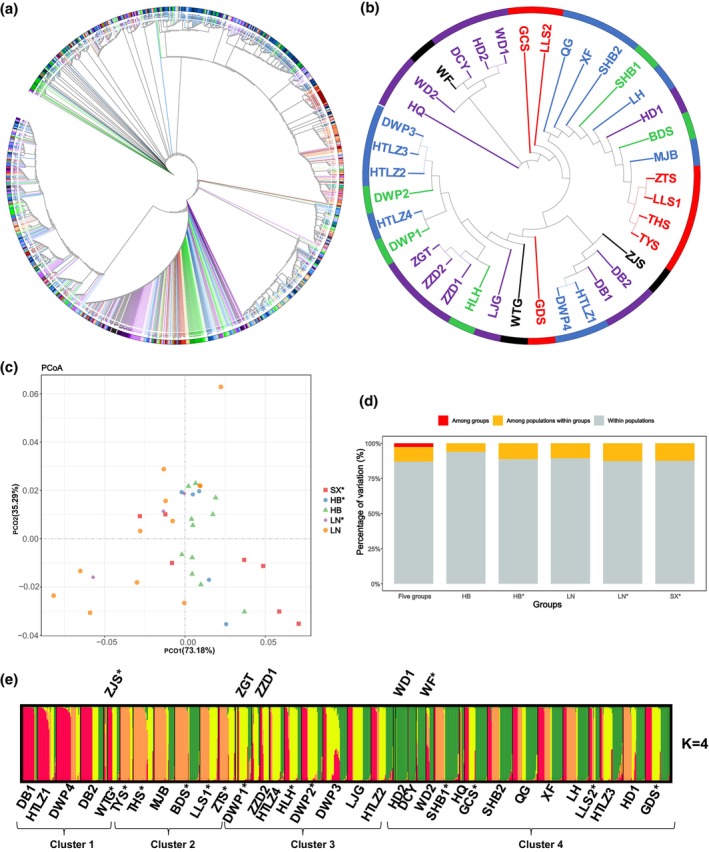
Genetic structure based on nad5‐1 marker. (a, b) The NJ tree (red, green, blue, black, and purple represent SX*, HB*, HB, LN*, and LN group, respectively, different transparencies represent different populations), (c) PCoA, (d) AMOVA, and (e) STRUCTURE results of 873 individuals (38 populations).

AMOVA generated the population structure based on the two markers, matR and nad5‐1. The results showed that 0.52% and 2.65% of the variation occurred between groups, 2.98% and 10.41% of the variation occurred between populations, while 96.50 and 86.95% of the variation occurred within populations for the matR (Figure 4d, Table S5) and nad5‐1 markers, respectively (Figure 5d, Table S6), suggesting that the variation of the five groups occurred mainly within populations and the nad5‐1 marker supports a stronger population structure. The higher between‐population variation was found for the nad5‐1 marker, the comparison of between‐population variation within groups was LN* > SX* > HB* > LN > HB, indicating that the SX* group had the higher degree of variation between populations (Figure 6d, Table S6).

To accurately reveal the population structure, the NJ‐tree, PCoA, and STRUCTURE were generated from the sequence data of the matR and nad5‐1 markers (Figures 5 and 6). Based on the matR marker, the results of the NJ tree (35 populations, 796 individuals, classified by groups) (Figure 5a) showed eight similar topologies and five groups were sorted in a fixed manner, indicating that there was a fixed genetic relationship among the five groups, and their relationship with ancestral status was as follows: SX*‐HB*‐HB‐LN*‐LN. The degree of dispersion of the LN populations in the NJ tree (Figure 5b) was greater than that of HB, suggesting that there was greater variation among populations within the LN group. The discrete distribution of the SX* populations (Figure 5b) indicates that there is also high variation among the populations. The PCoA results (Figure 5c) showed that the distribution of all populations was relatively concentrated, suggesting that the genetic differences among all populations at this locus were small. STRUCTURE analysis (Figure 5e) showed that the optimal number of the cluster was *K* = 4. We found that the proportions of the four ancestral components were almost equal in all populations, suggesting that there were only minor differences in genetic background between all populations at this locus. Based on the marker nad5‐1, the NJ‐tree (38 populations, 873 individuals, classified by groups) showed a complicated result and the difficulty of finding a genetic regularity of five groups (Figure 6a). The NJ‐trees classified based on the STRUCTURE results (Figure S1) also showed the same result. The LN populations were scattered in four topologies (Figure 6b), indicating that the LN group with high variation among populations was the same as that of the matR marker. The high variation between populations within the SX* group is also reflected in the NJ‐tree (Figure 6b). The PCoA results (Figure 6c) showed that the distribution of all populations was relatively scattered, suggesting that there was a large genetic difference between all populations at this locus. STRUCTURE analysis revealed *K* = 4 (Figure 6e), which corresponded to the value of the matR marker. All populations showed higher heterozygosity in the ancestral component at this locus, indicating that there was a large difference in genetic variation between all populations at this locus.

### 3.3 Haplotype analysis

**FIGURE 7:**
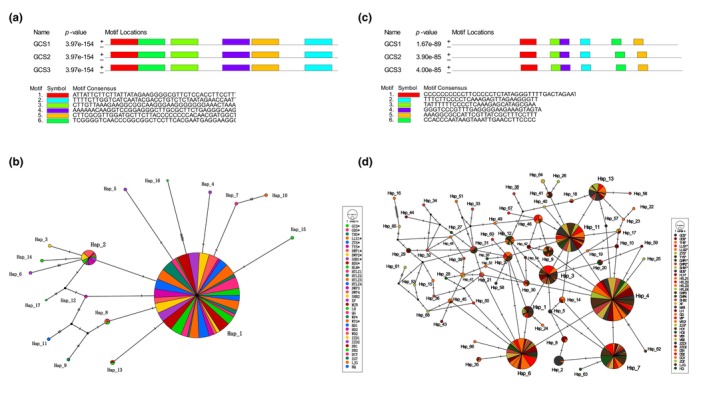
Haplotype network based on two markers. Six motif locations on matR (a) and nad5‐1 (c) markers, haplotype network based on six motif locations on matR (b) and nad5‐1 (d) markers.

A total of 17 haplotypes (27 bp) (Table S7) were identified based on six domains of the matR markers (Figure 7a). The haplotype distribution of 35 populations showed their genetic background (Figure 2), and the genetic association of all haplotypes was shown in the TCS network (Figure 7b). Hap‐1 was the most conservative and occupies a dominant position in all populations, indicating that Hap‐1 was the original(dominant) haplotype, followed by Hap‐2, which is present in most populations (10/35). Almost all other haplotypes (13/17) were private haplotypes belonging to a single population. There was no specific pattern of haplotype distribution in the five groups, and the distribution patterns of haplotypes in different populations provide limited available information for tracing plantations (Figures 2 and 7b, Table S7).

A total of 68 haplotypes (34 bp) (Table S8) were identified based on six domains of the nad5‐1 marker (Figure 7c). The different genetic backgrounds of the haplotypes of 38 populations (Figure 3) and the complex genetic relationships between the 68 haplotypes (Figure 7d) indicate that the nad5‐1 marker exhibits a high degree of variation. Hap‐4, hap‐6, and hap‐11 occur in almost all populations, especially hap‐4, suggesting that hap‐4 was the ancestral (dominant) haplotype, followed by hap‐7, hap‐3, and hap‐13, which occur in most populations. Almost all other haplotypes (40/68) were private haplotypes belonging to a single population, and the relationship between the 68 haplotypes showed a complicated network. There was a special pattern of haplotype distribution in five groups, hap‐4 and hap‐11 were the dominant haplotypes of SX*, HB*, and HB groups. The LN* and LN groups were dominated by hap‐7 and hap‐4. The diverse and regular haplotype distribution structures of 38 populations provide much valuable information for the traceability of the germplasm of plantation forests (Figures 3 and 7d, Table S8).

## 4 DISCUSSION

### 4.1 Genetic diversity, genetic structure, and haplotype characteristics of five groups

Both matR and nad5‐1 were neutral loci (Tables S2 and S3), which is consistent with the conservative variation of mtDNA (Held & Patel, 2020), which could provide reliable results for our research. Comparison of the genetic diversity of five groups at these two loci revealed similarities (Figure 4). As the conservatism of the variants at the matR site was higher, we took the genetic diversity of the matR site as a reference, which showed HB* > HB > SX*, LN > SX* > LN* (Pi) (Figure 4a). This was similar to our previous results (HB > HB* > SX*, LN* > LN > SX*) based on SSR markers (Zhou et al., 2022). The common point was HB > SX* and LN > SX*, showing that HB and LN plantings again have higher genetic variation.

The results of AMOVA, NJ‐tree, PCoA, and STRUCTURE consistently showed that the differences in the genetic background of all populations at these two loci were nad5‐1 > matR (Figures 5 and 6). The NJ‐tree showed the stationary lineage relationship of five groups (Figure 5a), suggesting SX* natural populations with an identical nuclear position in northern China, confirming our previous results (Zhou et al., 2022). Thirty‐eight populations of Chinese pine were categorized into four clusters based on matR and nad5‐1 loci (Figures 5e and 6e), which was inconsistent with the results based on SSR markers (six clusters), the consistent contents were as follows: SX* and LN showed large differences in genetic structure within groups, while those of HB showed little difference in genetic structure (Figures 5 and 6). The genetic structure of SX* and LN varied greatly within groups, suggesting that the genetic background of natural forests of different mountain lines in Shanxi is quite different (Wu et al., 2018; Zhou et al., 2022), and the germplasm sources of LN were more complex, it was not a single germplasm source. Within the HB group, there were only minor differences in genetic structure, suggesting that the germplasm sources of HB were relatively simple.

The results of haplotype analysis also showed that there were multiple dominant haplotypes in different populations in the SX* and LN groups, and the haplotype structure of the HB group was uniform, which was consistent with the results of genetic structure (Figures 2, 3 and 6). We firmly believe that there are great differences in the genetic background of Chinese pine natural forest in different geographical mountains of Shanxi, the germplasm sources of LN are more complex, and the germplasm sources of HB are relatively simple.

### 4.2 The exact germplasm source of HB and LN planting population

Analysis of mtDNA haplotype structure of species can effectively trace the germplasm source (Freeman et al., 2024). The distribution of haplotypes in the different populations (Figures 2, 3 and 7, Tables S7 and S8) refers to the phylogenetic relationship of all individuals and the cluster relationship based on the population (Figures 5 and 6), from which the germplasm sources of the plantation can be inferred. The fixed lineage relationship among the five groups was SX*‐HB*‐HB‐LN*‐LN (Figure 5a), which showed that the SX* group had an absolute core position in North China consistent with previous research results (Zhou et al., 2022). Twenty‐one haplotypes (nad5‐1) could provide valuable information for germplasm traceability of plantations, and the other seven haplotypes exist in some group privately (Table S8‐2).

**FIGURE 8:**
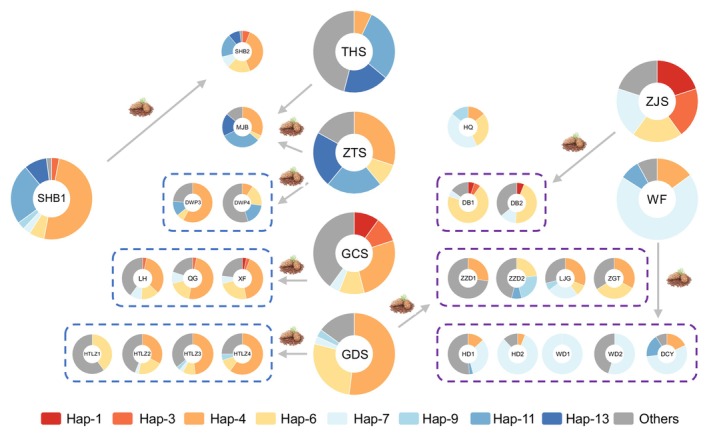
Exact germplasm sources of HB and LN plantations, and the haplotype structure of related populations.

Among the HB plantation populations, there were only minor differences in genetic structure (Figures 5 and 6) and a similar distribution pattern of haplotypes (Figures 2, 3 and 7), suggesting that the background of HB germplasm sources was relatively simple. SHB2 and SHB1* exhibited near‐identical haplotype distribution patterns (Table S8). Their genetic topologies and distances were closely aligned (Figures 5b and 6b), consistently grouping them into the same subgroup (Figures 4e and 5e). Geographically, the two populations were also adjacent (Figures 2 and 3, Table S1). These observations strongly suggest that the germplasm of SHB2 originated from SHB1* (Figure 8), further validating the accuracy of utilizing haplotype distribution, genetic distance, and ancestral composition to trace plantation germplasm sources. Hap‐6, the dominant haplotype in HTLZ1, comprises a significant portion of GDS* (9/33). The close genetic distance between HTLZ1 and GDS* (Figure 6b) suggests that the germplasm sources of HTLZ1 likely originated from GDS* (Figure 8). Hap‐4 dominated in HTLZ2‐4 as well as in several natural populations like GDS*(17/33), GCS*(8/31), ZTS*(7/21), LLS2*(5/12), SHB1*(17/33), DWP1*(9/17), DWP2*(9/23), HLH*(5/21), and BDS*(10/34) (Figure 3, Table S8), indicating HTLZ2‐4 might originate from these natural populations. According to our field investigations, SHB1* and BDS* had small sizes, limiting seed supply for plantations. DWP1‐2*, which had a similar age to HTLZ2‐4, was unlikely a source as it could be mistaken for a natural forest. Hap‐6, the secondary haplotype in HTLZ2‐4, was prevalent in GDS* (Figure 3, Table S8). The topological position of GDS* as an ancestor in relation to HTLZ2‐4 (Figure 6b) strengthened the case for GDS* as the likely source of HTLZ2‐4. Our findings confirmed that GDS* was most likely the germplasm source for HTLZ1‐4 (Figure 8). The close geographical proximity of HTLZ1‐4 and their shared germplasm source further validates our tracing method. Based on hap‐4 and hap‐11 (Figure 3, Table S8), it was deduced that DWP3 likely originated from ZTS* (Figure 8), and the neighboring genetic distance between them provided strong evidence for this (Figure 5b). Hap‐4, hap‐6, and hap‐11 were the key haplotypes for DWP4, mirroring ZTS*. Furthermore, GDS* was neighboring to DWP4 (Figure 6b) and represented its ancestral population (Figure 5b). It has been hypothesized that both ZTS* and GDS* could be the germplasm sources of DWP4. The germplasm sources of DWP4 and DWP3 should be the same, and the most likely germplasm sources of DWP4 should also be ZTS* (Figure 8). The distribution pattern of haplotypes of MJB closely resembled that of ZTS* and THS* (Table S8), pinned in the same topology with the close genetic distance (Figures 5b and 6b) and fixed in the same subgroup (Figures 5e and 6e). It was difficult to determine which of the two natural forests was more similar to the genetic background of MJB. We hypothesize that both ZTS* and THS* could be the germplasm sources of MJB (Figure 8). XF, LH, and QG were clustered in the same subgroup, sharing a consistent ancestral component and similar genetic topology with close distances (Figures 5b and 6e). Their haplotype patterns overlapped, with hap‐4, 11, 6, 13, and 7 as the main haplotypes (Figure 3, Table S8). GCS* exhibited a comparable haplotype distribution pattern to QG, XF and LH. Based on genetic distance and ancestral components, GCS* took the ancestral position of the subgroup containing QG, XF, and LH, suggesting their germplasm sources likely originated from GCS* (Figure 8).

The LN plantation populations displayed significant variations in genetic structure (Figures 4 and 5) and haplotype distribution (Figures 2, 3 and 7), indicating a complex germplasm background. The haplotype distribution patterns of HD1, HD2, WD1, WD2, and DCY were identical, dominated by hap‐7, matching only WF* (Figure 3, Table S8). Their close genetic relationship (Figures 5b and 6b) and similar genetic lineage (Figures 5e and 6e) suggest the five plantations' germplasm originated from WF*. Given WF*'s size, tree height, favorable location, and long‐term management, we infer that WF* supplied germplasms to HD1, HD2, WD1, WD2, and DCY (Figure 8). The NJ‐tree (Figure 6b) and genetic lineage analysis (Figure 6e) strongly suggest that the genetic material of DB1, DB2, and ZJS* is closely related, pointing to ZJS* as the source of germplasm for these plantations. Due to the limited sample size of the ZJS* population, we were unable to find strong evidence in favor of the haplotype distribution model. Based on the distribution of haplotypes, we could not exclude that GDS* and GCS* provided germplasm for DB1 and DB2, but we did not find this evidence in genetic distance and genetic ancestry. The short geographical distance between DB and ZJS* facilitated germplasm allocation (Figures 2 and 3, Table S1). With its convenient location and sizable population, ZJS* was an ideal source of germplasm for the local area, including DB1 and DB2 here (Figure 8). ZZD1, ZZD2, LJG, and ZGT were in the same topology with the same genetic distance (Figures 5b and 6b) and are fixed in the same cluster with a similar lineage (Figure 6e), indicating minimal differences in their genetic backgrounds. GDS* and WTG* occupied an ancestral position alongside ZZD1, ZZD2, LJG, and ZGT (Figure 6b), and these four plantations share a similar haplotype distribution pattern with GDS* and WTG*, suggesting that GDS* and WTG* may be the germplasm of these four plantations. However, our investigation reveals that WTG*, due to its remote location, small population, poor growth, and limited seed production, is unlikely to provide sufficient germplasm for plantation construction. Then, we speculated that the germplasms of ZZD1, ZZD2, LJG, and ZGT all likely originate from GDS* (Figure 8). HQ was nailed the priority position of the NJ tree (Figures 5b and 6b), and it was difficult to judge the origin of its germplasms according to its haplotype structure, genetic distance, and genetic lineage. We consulted HQ's reforestation archives and found that most of the germplasm came from the Xingcheng Seed Plantation (Chinese pine seed plantation in northern China), which was selected from excellent trees throughout Liaoning Province. The complex germplasm background of HQ brought challenges for the traceability of germplasm, and we could only laboriously determine its germplasm sources here (Figure 8).

To summarize, almost all HB populations originated from SX* (GDS*, ZTS*, GCS* and THS*), which resulted in homogeneity of the genetic background of HB populations. The provinces of Shanxi and Hebei are geographically close to each other, which facilitates the allocation of germplasm. Most of the germplasm of the LN plantations originated from LN* (ZJS* and WF*), the other part from GDS* (SX*), which led to large differences in the genetic structure within the LN group. The results are consistent with our previous speculation (Zhou et al., 2022).

## 5 CONCLUSION

In this study, we revealed the genetic diversity, genetic structure and haplotype traits of the major natural and artificial populations of Chinese pine in northern China using two mtDNA markers, and also accurately identified the germplasm sources of HB and LN plantations for the first time. We reconfirmed the core position of natural SX* populations in the Chinese pine populations of northern China. The genetic diversity of HB and LN plantations was higher than that of natural SX* populations, and there was a large difference in genetic background within the groups of SX* and LN, and the reverse was true for HB. More importantly, we completed the task of “point by point” tracing of the HB and LN plantings. We surmised that almost all HB populations originated from SX* (GDS*, ZTS*, GCS*, and THS*), which resulted in the homogeneity of the genetic background of HB populations. Most of the germplasm of the LN plantings originated from LN* (ZJS* and WF*) and the other part from GDS* (SX*), resulting in large differences in genetic background within the LN group.

Overall, our results provided a reliable theoretical basis for the scientific allocation, management, and utilization of Chinese pine populations in northern China and promoted the establishment of high‐quality Chinese pine plantations.

## AUTHOR CONTRIBUTIONS

**Biao Zhou:** Conceptualization (equal); data curation (equal); formal analysis (equal); investigation (equal); methodology (equal); resources (equal); software (equal); supervision (equal); validation (equal); visualization (equal); writing – original draft (equal); writing – review and editing (equal). **Zijie Zhang:** Resources (supporting). **Hongjing Zhang:** Resources (supporting). **Yupeng Li:** Resources (supporting). **Yanguang Ma:** Resources (supporting). **Shubin Zhang:** Resources (supporting). **Shihui Niu:** Funding acquisition (equal); project administration (equal); writing – review and editing (lead). **Yue Li:** Conceptualization (lead); resources (lead); supervision (equal); writing – review and editing (lead).

## FUNDING INFORMATION

This work was supported by the “Biological Breeding‐Major Projects (2023ZD040580404)” and the “Key Research and Development Program of Hebei Province (21326351D)”.

## CONFLICT OF INTEREST STATEMENT

No conflict of interest exists in the submission of this manuscript, and the manuscript is approved by all authors for publication.

## Supporting information


Data S1

Figure S1

Tables S1–S8

## Data Availability

The raw sequencing data have been saved in “Raw sequencing data.”
