# Supplementary figures and images for: “Point by point” source: The Chinese pine plantations in North China by evidence from mtDNA
Source: Ecol Evol. 2024 Jun 19;14(6):e11570. doi: 10.1002/ece3.11570 (PMC11185947; doi:10.1002/ece3.11570)

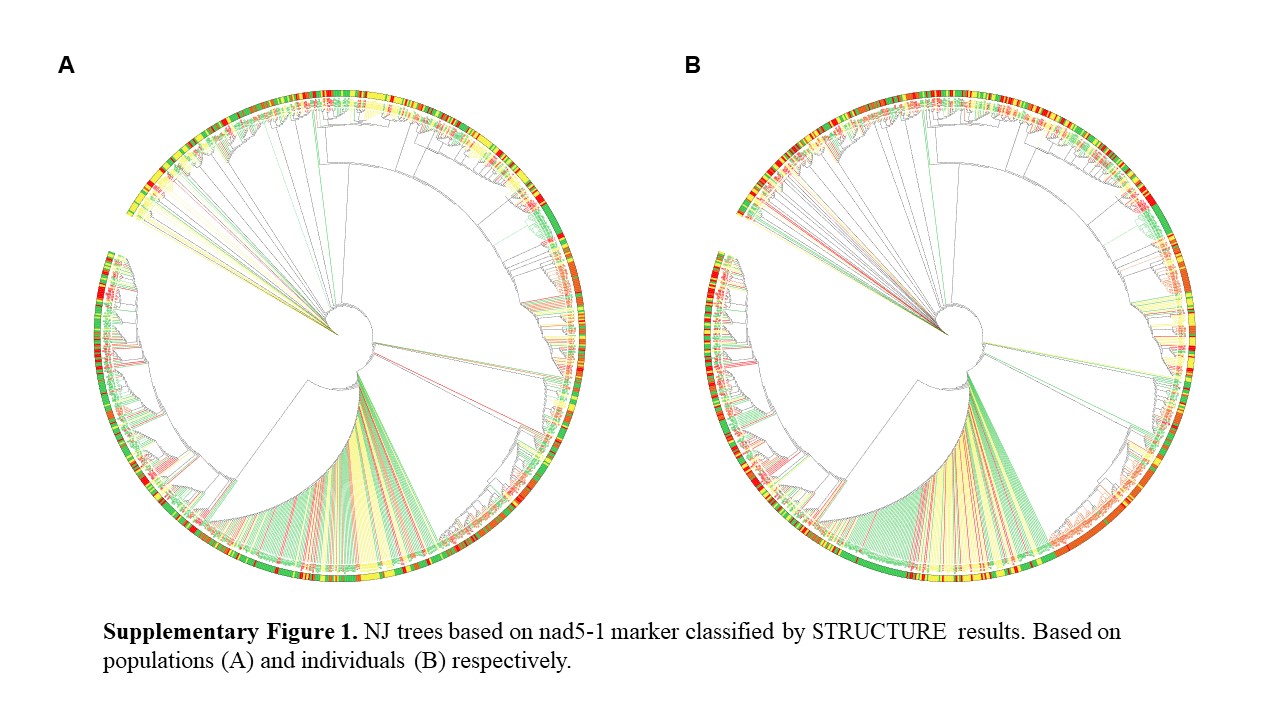

Supplement: Supplementary file 2 — Figure S1 [file ECE3-14-e11570-s002.jpg]
